# The prevalence, risk factors, and screening measure for prediabetes and diabetes among Emirati overweight/obese children and adolescents

**DOI:** 10.1186/s12889-015-2649-6

**Published:** 2015-12-24

**Authors:** Elham Al Amiri, Mona Abdullatif, Abdishakur Abdulle, Nibal Al Bitar, Elham Zaki Afandi, Monira Parish, Gassan Darwiche

**Affiliations:** Department of Pediatrics, Al Qassimi Hospital, Ministry of Health, P.O.Box: 3500, Sharjah, United Arab Emirates; Department of Medical Education, Dubai Health Authority, Dubai, United Arab Emirates; Department of Internal Medicine, College of Medicine and Health Sciences, UAE University, Al Ain, United Arab Emirates; Department of School Health, Ministry of Health, Sharjah, United Arab Emirates; Department of Internal Medicine, Skane University Hospital, Lund University, Lund, Sweden

**Keywords:** Diabetes mellitus, Prediabetes, Overweight, Obese, Children, Adolescence

## Abstract

**Background:**

The aim of the study was to estimate the prevalence of prediabetes and type 2 diabetes (T2D) among overweight/obese children and adolescents using different diagnostic/screening methods in comparison.

**Methods:**

We recruited overweight/obese Emirati students; grade 6–12 (age 11–17 years) from 16 government schools in Sharjah (UAE). Anthropometric, demographic, and clinical history data was measured by standard methods. Body mass index (BMI) was categorized according to BMI percentile charts for age and sex – CDC. Capillary fasting blood glucose (FBG) and glycated hemoglobin (HbA1c) were measured by finger prick test, followed by confirmatory oral glucose tolerance tests (OGTT) and venous HbA1c for students with abnormal capillary FBG and/or HbA1c.

**Results:**

Of a total of 1034 participants (45 % females) median age 14.7 years, 443 (43 %) students had abnormal screening results. The prevalence of prediabetes and T2D was 5.4 % and 0.87 %, respectively, based on OGTT (gold standard). HbA1c showed a considerable discrepancy regarding the prevalence of prediabetes (21.9 %), but not diabetes. There was a statistically significant difference in the BMI Z-scores between the three different groups of students showing normal glycemic testing, prediabetes and T2D (*p* = 0.041). Univariate logistic regression analysis showed that glycemic status was significantly associated with family history of T2D first-degree relatives [OR 1.87: 95 % CI: 1.04–3.36; *P* = 0.036], parents employment [OR 1.79: 95 % CI: 1.06–3.02; *P* = 0.029] and levels of triglycerides [OR 2.28: 95 % CI: 1.11–4.68; *P* = 0.024].

**Conclusions:**

The prevalence of prediabetes and diabetes was high among overweight/obese Emirati children and adolescents. The numbers for prediabetes were considerably higher when using HbA1c as compared to OGTT. Overall adiposity, family history of T2D, employment and high levels of triglycerides were risk factors associated with abnormal glycemic testing.

## Background

The prevalence of type 2 diabetes (T2D) is rapidly increasing and has become a major public health challenge worldwide among the adult populations and, to a lesser extent, among children and adolescents [1, 2]. These new trends are mostly attributable to a high prevalence of childhood overweight and obesity – a phenomenon that is being recognized as a future harbinger for deleterious health outcomes. Obese children and adolescents are at a higher risk for glucose intolerance, T2D, early signs of insulin resistance, and cardiovascular diseases presumably due to environmental and genetic factors [3, 4].

Since the discovery of oil in the Arabian Gulf countries, there has been a rapid socioeconomic transition towards an affluent life style leading to a new trend of obesity and associated diseases, including diabetes, to an epidemic level [5, 6]. Several studies have shown a high prevalence of T2D among children in Oman [7], Saudi Arabia [8], Kuwait [9, 10] and the UAE [11]. In the UAE, some hospital based studies showed that about 10 % of children diagnosed with diabetes suffer from T2D [12]. Moreover, 2–3 % of all deaths in the last ten years are attributable to diabetes [13]. This may be explained, in part, by compounding factors of diminished exercise, increased weight, glucose intolerance and consequently overt diabetes mellitus [14]. The American Diabetes Association (ADA) recommended the use of HbA1c levels for prediabetes screening in both children and adolescents [15]. However, the usefulness of HbA1c is currently under debate [16]. The aim of this study was to estimate the prevalence of prediabetes, T2D, and associated risk factors among overweight and obese Emirati children and adolescents in the UAE. A secondary objective was to compare the screening results from different diagnostic methods.

## Methods

### Ethical considerations

The Ethics Committee of the Ministry of Health, UAE, approved the study. Students were included in the study only after they gave assent and the parents gave permission. Data were collected, revised and pseudonymized by the principal investigator and entered into the study database by trained staff. For ethical reasons, we referred all students with abnormal glucose levels to their family physician.

### Subjects

The School health Program in the UAE have a national physical examination screening program including measuring height and weight and charting BMI on growth charts for all students. The procedure is standardized and conducted by trained school nurses. Instructions, like students being bare-foot and in minimal clothing, are given before weight is measured with electronic scales that are calibrated periodically by bio-medical engineering department. List of due date for calibration of electronic scales are maintained in the school health department. In this cross sectional study, we invited all overweight/obese Emirati students from grade 6–12 aged 11–17 years from the records of all public government schools in Sharjah. Of the total (20), only 16 schools have had complete student data in terms of height, weight and calculated Body Mass Index (BMI). Inclusion criteria were UAE national, children and adolescents who were either overweight or obese according to BMI percentile (as defined below). Exclusion criteria were children with known type 1 diabetes, thalassemia major, sickle cell anemia and children on steroid treatment.

### Testing procedures

In the first phase, a standard questionnaire was sent to the parents along with information sheet and consent form. All school visits were accomplished in five weeks between April and May 2011. Data was collected through interviews using questionnaires, physical examinations with collection of anthropometric data and blood tests as described below. The enrolled participants were asked to come to the school clinic in the morning after 10 hour overnight fast. Weight, height, blood pressure (BP) and waist circumference (WC) were measured as per the below mentioned methods. Presence of acanthosis nigricans was checked by the principal investigator by examining the neck fold. Further, a finger prick sample was obtained for capillary fasting blood glucose and capillary HbA1c. If fasting blood glucose was abnormal (≥100 mg/dl or ≥ 5.6 mmol/l) the participant was asked to come back on another (often the following) morning for a second fasting test for confirmation. Participants with abnormal capillary HbA1c (≥5.7 % or ≥ 38 mmol/mol), and/or abnormal second fasting capillary blood glucose (≥100 mg/dl or ≥ 5.6 mmol/l) were eligible for a second phase [15]. Self-reported type 2 diabetes was included.

In the second phase, parents of the children with abnormal screening glucose levels/ HbA1c were contacted and were given another appointment within two weeks. Blood samples were analyzed in a reference laboratory at Al Qassimi hospital, Sharjah. On the visit day, a sample of venous blood (10 mL) was collected for HbA1c, fasting lipid profile (total cholesterol, triglycerides, HDL- and LDL cholesterol). Additionally, a standard oral glucose tolerance test (OGTT) with a dose of 1.75 g glucose per kilogram of body weight (up to a maximum of 75 g) after a 10 hour overnight fast was done. OGTT was performed during standardized conditions. Participants were instructed to live as normal as possible in respect to diet and physical activity the days before the OGTT. The test was postponed to another day in the event of ongoing infection. They were also instructed not to exercise and to abstain from food, fluids (except water) and tobacco from 10 pm the night before the test. The 10 hour fasting was confirmed by asking both the participants and their parents. Prediabetes (fasting glucose 100–125 mg/dl equivalent to 5.6–6.9 mmol/l or 2-h glucose 140–199 mg/dl equivalent to 7.8–11.0 mmol/l) and diabetes (fasting glucose ≥126 mg/dl equivalent to ≥7.0 mmol/l or 2-h glucose ≥200 mg/dl equivalent to ≥11.1 mmol/l) was defined by glucose levels obtained during the OGTT according to the American Diabetes Association (ADA) guidelines [15]. Based on the ADA guidelines for HbA1c prediabetes was defined as HbA1c between 5.7 % – 6.4 % (38 – 47 mmol/mol) and diabetes as HbA1c ≥ 6.5 % (≥48 mmol/mol) [15]. Dyslipidaemia was defined as total cholesterol > 5.2 mmol/l; triglycerides >1.7 mmol/l; HDL below the normal reference range (1.04–1.55 mmol/l); LDL >3.9 mmol/l.

### Questionnaire

A self-administered questionnaire was sent to the parents through their children. The questionnaire asked about parents’ education level and employment status, consanguinity between the parents as well as information regarding the child’s health condition. In addition, information was collected regarding the child’s clinical history i.e., blood disorders such as sickle cell anemia or thalassemia, medication, family history (diabetes, hypertension and dyslipidemia), and symptoms associated with hyperglycemia (excessive thirst and drinking, frequent urination, recent weight loss, fatigue and recurrent thrush or skin infections). Further, exercise habits using physical activity score based on different levels of physical leisure activity [no activity, activity (1 time/week), regular activity (1–2 times/week), regular activity (3–5 times/week), and regular daily activity] were recorded using a validated questionnaire for physical exercise taken from the National Diabetes Register (NDR), [17]. NDR is one of Sweden’s national quality registers operated by the Swedish Society for Diabetology (SFD) on behalf and with the support of local authorities and the Swedish National Board of Health and Welfare. The questionnaire is used for adults as well as children and adolescents (through SWEDIABKIDS) to facilitate systematic quality work at the participating care units.

### Weight, height, and waist circumference measurements

All measurements were taken by a trained nurse. Children were weighed without shoes or heavy clothing to the nearest 0.1 Kg, and their height was measured to the nearest 0.1 cm on a calibrated scale with attached stadiometer (Seca stadiometer and weighing scale, Seca, Hamburg, Germany). A standard measuring tape was used to measure WC at a point right above the iliac crest on the midaxillary line at minimal respiration and the results were rounded to the nearest 1.0 cm. In all cases, two separate measurements of weight, height and WC were collected and averaged for analysis. We used Epi info software to calculate BMI as the ratio of weight to height squared (kg/m2) and BMI percentiles according to percentile charts for age and sex from the Centers for Disease Control and Prevention (CDC), subsequently, children’s weights were classified as underweight: BMI < 5th% ile, normal weight: BMI ≥ 5th to <85th% ile, overweight: BMI ≥ 85th to < 95th% ile, and obese: BMI ≥ 95th% ile [18].

### Blood pressure measurements

BP was measured using calibrated Omron M6 IntelliSense (Healthcare, Kyoto, Japan) automatic BP monitors with appropriate cuff size [19]. Prior to taking BP readings all students were instructed to rest for at least 10 minutes in an air-conditioned environment. Measurements were taken two times on the right arm with short intervals between readings, and the average of BP readings was calculated and used for analysis. Blood pressure was categorized as normotensive [systolic blood pressure (SBP) and diastolic blood pressure (DBP) <90th percentile (% ile)]; Prehypertension (high normal) [SBP and/or DBP ≥ 90^th^ <95^th^ ile]; Hypertension [SBP and/or DBP ≥95^th^ ile]; for age, sex, and height [20].

### Biochemical measurements

Capillary blood glucose was measured by glucometers (Performa, Roche, Germany). Capillary HbA1c was measured by a portable disposable multi-test HbA1c system (A1c Now+, Bayer, Germany). The analysis was quality assured using sample selections of HbA1c values for comparison at the laboratory at Rashid Centre for Diabetes and Research (RCDR), (Roche, Tina-quant HbA1cDx Gen.assay). The RCDR laboratory participates in the (EQA) program (proficiency test program). Analyses of plasma glucose, total cholesterol, triglyceride, HDL, LDL and plasma HbA1c were made using a standard chemistry analyzer (Dimension RXL max, Siemens, Germany), which gives HbA1c values according to NGSP % HbA1c.

### Statistical evaluation

Statistical analyses were performed using STATA version 12.0 for Windows. Variables were tested for normality both visually and statistically and most of the variables lacked normal distribution. Accordingly non-parametric tests were used and the study population was described using median values with quartiles (q1 to q3). To compare continuous and categorical variables, we used Kruskal–Wallis and Fisher’s exact test, respectively. BMI Z-score were used to explore the association between BMI and diabetes or prediabetes. Univariate logistic regression analysis was used to estimate the odds associated with prediabetes and diabetes for selected risk factors. P–value < 0.05 was considered statistically significant.

## Results

### Clinical features of the study population

**Fig. 1:**
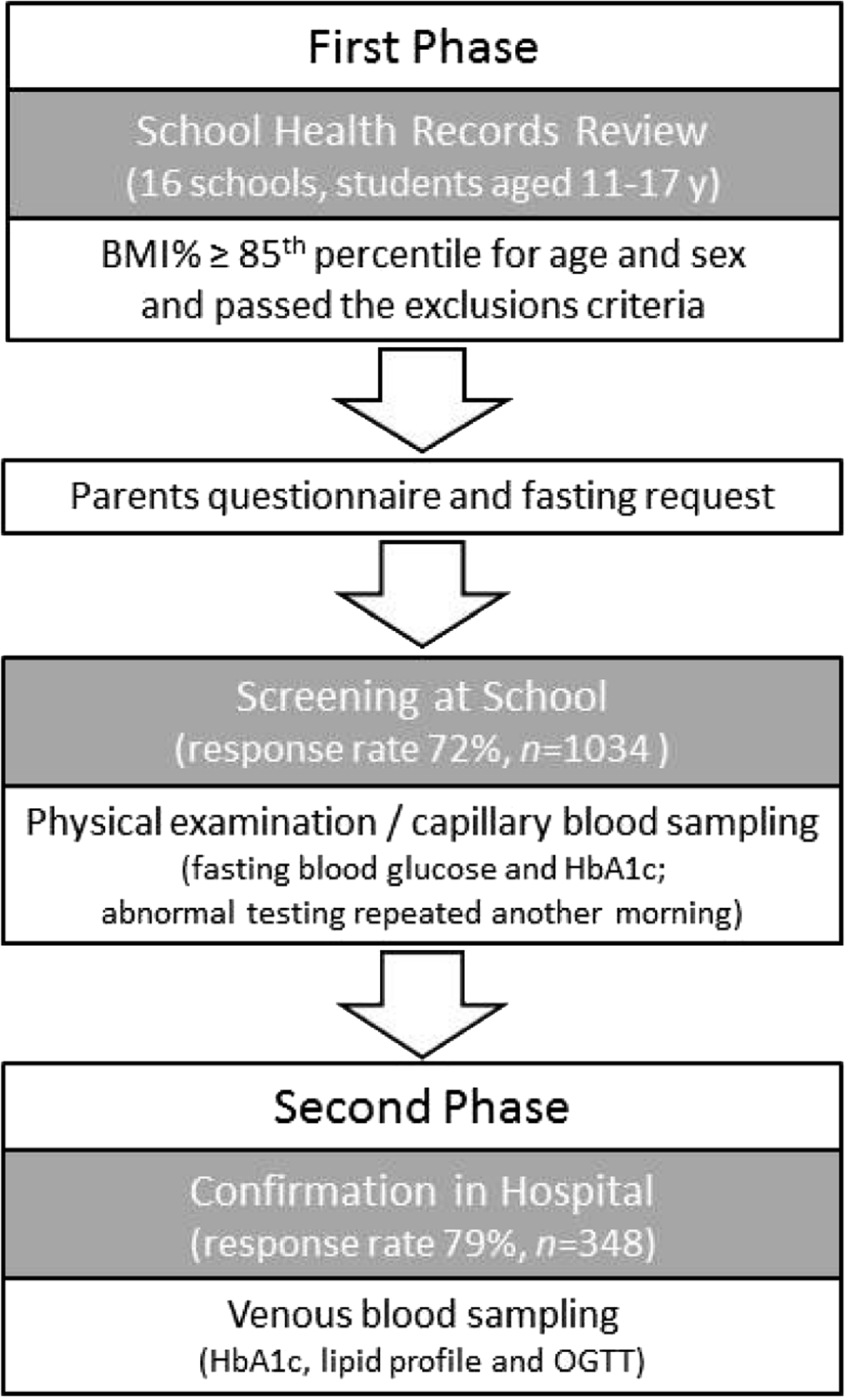
Flowchart showing the study design. From the school health records of 16 public schools in Sharjah (the overall cohort *n* = 7088 students), 1436 Emirati students aged 11–17 years were identified according to the inclusion criteria. The response rate was 72 % (n = 1034) in the first phase and 79 % (n = 348) in the second phase of confirmation testing

From the school health records of 16 schools (the overall cohort n = 7088 students), 1436 Emirati students aged 11–17 years were identified, who were either overweight or obese according to our inclusion/ exclusions criteria. All these students were invited to participate in the study. Of the total, 1034 completed the consent process and were enrolled for screening with a response rate of 72 % (Fig. 1). The median age of the study population was 14.7 (13.2–16.2) years, of whom 45 % were females.

Table 1 shows the characteristics of the participants stratified by sex. Although the prevalence of diabetes among first-degree relatives was higher among female students (*p* = 0.003) there were no differences in the incidence of abnormal glycemic testing between the groups (*p* = 0.43). Socio-demographic data indicated a relatively high percentage of consanguinity between the parents (32 %). The results also show a greater proportion of mothers who are unemployed (79 %) compared to the fathers (35 %). Only 16 % of the families had both parents working. Clinical features of the study population shows a distribution of 78 % obesity and 22 % overweight according to the BMI percentile charts for age and sex.

**Table 1:** Demographic and anthropometric data of the study population stratified by sex

Variables	Females (*n* =465)	Males (*n* =569)
	Median (q1 - q3)	Median (q1 - q3)
Age (years)	15.2 (13.4–16.4)	14.5 (13.1–15.9)
Height (cm)	156.5 (152.0–160.5)	163.5 (155.5–170.5)
Weight (kg)	76.3 (66.6–87.0)	81.3 (67.2–97.0)
BMI (kg/m^2^)	31.0 (28.1–34.8)	30.0 (26.8–34.0)
BMI (% ile)	97.7 (95.1–98.7)	98.0 (95.7–99.2)
Overweight (%)	24	20
Obese (%)	76	80
WC (cm)	92.0 (84.0–101.0)	98.0 (89.0–107.0)
Waist to Height ratio	0.59 (0.54–0.65)	0.60 (0.55–0.66)
SBP (mmHg)	114.0 (105.3–123.0)	120.0 (110.0–130.0)
DBP (mmHg)	70.0 (68.3–80.0)	70.0 (65.0–80.0)
Parental employment (%)		
Father working	63	63
Mother working	21	20
One or both working	70	71
Neither working	30	29
1^st^ degree relative with diabetes (%)	53	41
Abnormal glycemic testing (%)	6	7
Parental Consanguinity [n (%)]	140 (30)	177 (31)
Polyuria [n (%)]	33 (7)	35 (6)
Polydipsia [n (%)]	62 (13)	57 (10)
Weight loss [n (%)]	10 (2)	11 (2)
Fatigue [n (%)]	77 (17)	101 (18)
Recurrent Infections [n (%)]	42 (9)	33 (6)
Acanthosis [n (%)]	158 (34)	421 (74)

Median values with quartiles (q1–q3) are shown. *BMI* body mass index, *WC* waist circumference, *SBP* systolic diastolic pressure, *DBP* diastolic blood pressure

### Glycemic status and correlation to risk factors

Out of 443 children with abnormal screening for blood glucose and/or HbA1c levels, 348 children (79 %) showed up for the venous confirmation tests at the hospital laboratory, (Fig. 1). Using diagnostic screening practices based on the fasting blood glucose and/or the 2-h glucose levels obtained during the OGTT, the estimated prevalence of prediabetes and T2D in this study population was 5.4 % (56 cases out of 1034) and 0.87 % (9 cases out of 1034) respectively. The results showed poor agreement with the venous HbA1c methodology which generally pointed to a higher proportion of children with prediabetes (21.9 %) defined as HbA1c 5.7 – 6.4 % (38 – 47 mmol/mol), [15]. In comparison with the OGTT (gold standard), the HbA1c method showed a sensitivity of 0.52 and a specificity of 0.34 for the diagnosis of prediabetes in children and adolescents. However, given the small sample size, it was not possible to compare the two methods for sensitivity and specificity for the diagnosis of diabetes. Also, capillary HbA1c values showed poor agreement with capillary fasting blood glucose values, with only 11 % of cases showing consistency between elevations. Confirmatory results comparing the three glycemic testing methods are presented in Table 2.

**Table 2:** The prevalence of normal, prediabetes, and diabetes according to the confirmatory glycemic testing methods, in comparison (*n* =348)

	HbA1c (mmol/mol)		FBG (mmol/L)		OGTT (mmol/L)	
	RV	%	RV	%	RV	%
Normal	< 38	34.0	< 5.6	87.3	< 7.8	91.9
Prediabetes	38 – 47	65.1	5.6 – 6.9	12.1	7.8 – 11.0	7.2
Diabetes	≥ 48	0.9	≥ 7.0	0.6	≥ 11.1	0.9

*RV* reference value

No significant correlation was seen between glycemic status and level of physical activity, presence of acanthosis nigricans or symptoms of diabetes, except for weight loss (*p* = 0.032). Family history of T2D first-degree relatives (*p* = 0.028) and high levels of triglycerides (*p* = 0.019) were statistically correlated with abnormal glycemic testing based on fasting blood glucose and 2-h postprandial blood glucose (OGTT), (Table 3). There was a statistically significant difference in the BMI Z-scores between the three different groups of students showing normal glycemic testing, prediabetes and T2D (*p* = 0.041), as shown in Table 3. The specific groups that differed were diabetic students versus those with normal glycemic testing (*p* = 0.024). No significant correlation was shown related to age, sex, WC, Waist-to-Height ratio, systolic or diastolic blood pressure, parents’ employment, cholesterol levels, HDL or LDL. Table 4 show the results of logistic regression analysis of the association between abnormal glycemic status, based on fasting glucose and 2-h Glucose (OGTT), and selected factors. The results indicate that a family history of diabetes, parents being unemployed and high levels of triglycerides were independent risk factors for abnormal glycemic testing (*p* < 0.05). The prevalence of abnormal glycemic testing was 1.9 times more common among students with a family history of diabetes among first-degree relatives. Having both parents unemployed increased the risk for abnormal glycemic testing by 1.8 times compared to having one or both parents employed. Abnormal blood glucose testing was 2.28 times more common among students with high levels of triglycerides.

**Table 3:** Comparison of factors associated with diabetes in the sample population

Parameters		Normal (*n* = 967)	Pre DM (*n* = 56)	DM (*n* = 9)	*P* value
Age (years)		14.7 (13.2 – 16.2)	14.7 (13.0 – 15.9)	15.6 (15.0 – 17.7)	0.136
Sex (%)	Female	435 (94.3)	23 (4.9)	3 (0.65)	0.669
	Male	523 (93.2)	33 (5.8)	6 (1.1)	
BMI % ile (%)	Overweight	215 (95.6)	10 (4.4)	0	0.259
	Obese	748 (93.2)	46 (5.7)	9 (1.1)	
BMI Z-score		2.0 (1.7 – 2.3)	2.1 (1.8 – 2.4)	2.2 (2.1 – 2.4)	0.041
WC (cm)		95.0 (86.0 – 104.9)	96.0 (89.8 – 105.0)	104.0 (99.0 – 114.0)	0.096
Waist to Height ratio		0.6 (0.5 – 0.7)	0.6 (0.6 – 0.7)	0.6 (0.6 – 0.7)	0.108
SBP (mmHg)		118.0 (110.0 – 126.0)	120.0 (110.0 – 130.0)	116.0 (110.0 – 121.0)	0.452
DBP (mmHg)		70.0 (65.0 – 80.0)	70.0 (65.0 – 80.0)	74.0 (67.0 – 77.0)	0.873
Parental employment	Neither (%)	267 (91.1)	21 (7.1)	5 (1.7)	0.118
	One parent (%)	508 (94.9)	25 (4.6)	2 (0.4)	
	Both parents (%)	152 (94.4)	7 (4.3)	2 (1.24)	
1^st^ degree relative (%)	No	326 (94.8)	18 (5.2)	0.0	0.028
	Yes	280 (91.2)	22 (7.2)	5 (1.63)	
TG (mmol/L)	Normal	256 (84.1)	45 (15.1)	2 (0.7)	0.019
	High	31 (70.5)	11 (25.0)	2 (4.6)	
Cholesterol (mmol/L)	Normal	266 (82.5)	53 (16.5)	3 (0.9)	0.322
	High	21 (84)	3 (12.5)	1 (4.0)	
HDL (mmol/L)	Low	93 (79.5)	22 (17.1)	2 (1.7)	0.491
	Normal	168 (85.3)	28 (14.2)	2 (1.7)	
	High	19 (79.2)	5 (20.8)	0.0	
LDL (mmol/L)	Normal	272 (83.4)	51 (15.6)	3 (0.9)	0.187
	High	5 (62.5)	3 (37.5)	0	

Median values with quartiles (q1–q3) are shown. We used Kruskal–Wallis test to compare continuous variables; Fisher’s exact to compare categorical variables. Significance level = *p* < 0.05

**Table 4:** Univariate logistic regression analysis model of the association between abnormal glycaemic status based on fasting glucose and 2-h Glucose (OGTT), and selected factors

Variables	OR	% 95 CI	*P*-value
Age (11–14/ 15–17 years)	1.02	0.69–1.52	0.908
Sex (Female/ Male)	1.23	0.74–2.05	0.434
1^st^ degree relative with diabetes	1.87	1.04–3.36	0.036
Parental employment (One or Both/Neither)	1.79	1.06–3.02	0.029
Parents relatives (No/Yes)	1.22	0.71–2.09	0.474
BMI% (Overweight/ Obese)	1.58	0.79–3.15	0.196
Waist circumference (<102/ ≥102 cm)	1.38	0.83–2.31	0.217
Exercise (Activity ≥ 1 time/week/No activity)	0.89	0.53–1.48	0.646
Hypertension (No/ Yes)	1.33	0.66–2.68	0.430
Triglycerides (Normal/ High)	2.28	1.11–4.68	0.024
Cholesterol (Normal/ High)	0.91	0.30–2.74	0.859
HDL (Normal or High/ Low)	1.42	0.80–2.53	0.236
LDL (Normal/ High)	3.02	0.70–13.0	0.138

*P-*values <0.05 was considered as statistically significant

## Discussion

We report that the prevalence of prediabetes and T2D among overweight and obese children and adolescents is high based on OGTT as well as HbA1c, on which the HbA1c method show significantly higher rates for prediabetes but not diabetes. We also show that glycemic status among children is significantly associated with overall adiposity (BMI Z-score), family history of T2D, and levels of triglycerides.

The United Arab Emirates has one of the highest prevalence of diabetes in the world, but T2D in children has been considered as a rarity until recently. Existing data about T2D in paediatrics are scarce and usually in-hospital rather than community-based settings [21]. Obesity is an increasing health concern worldwide due to its association with diabetes, the metabolic syndrome and related health risks. Globally, childhood obesity has dramatically increased to reach epidemic proportions in the last decades raising concerns about an increased prevalence of T2D down the ages [2]. In the UAE, it has been reported that childhood obesity is as high as 40 % among school children [22].

In this cohort of Emirati overweight/obese children and adolescents, we estimated the prevalence of prediabetes to 5.4 % and T2D to 0.87 % based on traditional diagnostic screening practices (fasting plasma glucose and/or OGTT). These results can be compared with data from Kuwait where the prevalence of T2D among randomly selected children and adolescents was estimated at 34.9 per 100 000 children aged 6–18 years [10]. Between 1990 and 1998 12.5 % of all patients aged up to18 years with new-onset diabetes at Al-Ain general hospital were diagnosed as having type 2 diabetes. These patients were superobese and had a positive family history of T2D [23]. Our results are comparable with findings from Saudi Arabia among T2D population of which a prevalence of 0.12 % among children and 0.79 % among under-14-years children and younger adults 14- to 29-year was reported [24]. However, our figures are less than previously reported by Sinha and co-workers [25]. Still, the study population in the present study also included overweight children and adolescents where the probability of finding prediabetes and diabetes is likely lower than in a study population consisting of participants solely marked obese.

Previously endorsed screening guidelines has recommended children with body mass index ≥85th percentile and any two additional risk factors to be screened with a fasting plasma glucose (FPG) or a 2-hour glucose tolerance test (OGTT) every 2 years starting at age 10 years, or at onset of puberty [26, 27]. In 2010, the American Diabetes Association (ADA) published revised and modified diagnostic guidelines recommending that HbA1c tests also be used for diagnosing diabetes (HbA1c ≥ 6.5 % or ≥ 48 mmol/mol) and prediabetes (HbA1c = 5.7 %–6.4 % or 38 – 47 mmol/mol) in both adults and children [28]. However, these recommendations have been questioned as they were considered being based strictly on data from adult studies and lack any input from pediatric research. In fact, the HbA1c method has been claimed to represents a poor diagnostic tool in children and adolescents due to a relatively lower test performance compared with adults [29, 30]. Several studies published on the topic indicate that using adult cutoff points for HbA1c values to predict prediabetes or diabetes significantly underestimates the prevalence of these conditions in the pediatric and adolescent population [29–33]. Consequently a lower HbA1c cut-off point has been proposed for children. Not unexpectedly, the results from our study reveal a poor agreement between diagnostic screening based on fasting plasma glucose or OGTT and venous HbA1c (Table 2). However, while other studies have shown that HbA1c seem to underestimate the prevalence of T2D and prediabetes, our results indicate a higher proportion of children with prediabetes (21.9 %) using HbA1c. Also, capillary HbA1c values showed poor agreement with capillary fasting blood glucose values with only 11 % of cases showing consistency between elevations. Nevertheless, the design of the study using OGTT as a confirmatory test in cases of suspected diabetes and prediabetes based on capillary fasting blood glucose and capillary HbA1c, may have missed to identify a potential group of obese children and adolescents with prediabetes showing normal fasting glucose and normal HbA1c but eventually abnormal OGTT (IGT). These results raise some unanswered questions for the future. Does HbA1c overestimates the problem being less specific or is it rather a more sensitive test? Most likely the various diagnostic methods do not overlap but instead identify different groups of participants with prediabetes and diabetes [34, 35]. A follow-up study would be able to clarify which of the methods to be deemed most reliable in predicting progression to diabetes among these variety of prediabetic children and adolescents.

More than half of all participants diagnosed as prediabetic and all participants diagnosed as having diabetes had a first-degree relative with T2D. Additionally, 20 % of the prediabetes participants and about 50 % of diabetes have had elevated triglyceride levels as compared with 11 % in the normal glycemic status group. No significant correlations were observed with regards to age, sex, WC, Waist-to-Height ratio, levels of cholesterol, HDL or LDL, probably due to lower statistical power needed to detect significant statistical relations. However, in agreement with results from other studies, a family history of T2D first-degree relatives (*p* = 0.028) and high levels of triglycerides (*p* = 0.019) was statistically and significantly correlated with abnormal glycemic testing based on fasting blood glucose and 2-h postprandial blood glucose (OGTT), [5, 36]. In fact, abnormal glycemic testing was 1.9 times more common among students with a family history of diabetes among first-degree relatives and 2.28 times more common among students with high levels of triglycerides (Table 4). Furthermore, having both parents not working was related to 79 % higher prevalence of diabetes compared to having one or both parents employed. Although unemployment is often associated with economic inactivity, it may rather be an expression of economic independence and a sedentary lifestyle in a region with increased affluence linked to the diabetes epidemic. Relationship by blood is an important risk factor for type 2 diabetes to consider. Previous epidemiologic studies have shown that people with a family history of diabetes in first-degree relatives who are affected with diabetes are 2 to 6 times as likely to have the disease compared with people who have no affected relatives [37]. The United Arab Emirates has one of the highest prevalence of type 2 diabetes in the world and marriages between cousins are common, which could increase the risk of getting type 2 diabetes.

Hyperglycaemia has been associated with high levels of triglycerides. Improving glycemic control in individuals with moderate to severe hyperglycemia regardless of type of treatment is associated with improvement in lipid values [38]. Obesity has been stated to be the most important cause in the development of insulin resistance and it has been demonstrated that the critical determinant of insulin sensitivity is not the degree of obesity per se but the distribution of fat partitioning [39]. In our study the majority of subjects were obese and body mass index expressed as BMI Z-scores were correlated to abnormal blood glucose testing [25]. Actually, the median BMI percentile according to CDC definition in this study was 97.9 (95.6–99.0), which is quite high and explained by the fact that most of the participants were in the obese category (78 %). The reference population used to construct the CDC Growth Charts for children aged 2 years to 20 years is a nationally representative sample obtained from 5 national health examination surveys conducted by NCHS from 1963 to 1994. Survey-specific sample weights were applied to the national survey sample data to assure representation of the U.S. population according to age, gender, and racial/ethnic composition at the time the surveys were conducted. CDC promotes one set of growth charts for all racial and ethnic groups. Racial- and ethnic-specific charts are not recommended because studies support the premise that differences in growth among various racial and ethnic groups are the result of environmental rather than genetic influences [40]. No significant correlation was seen between glycemic status and level of physical activity, systolic or diastolic blood pressure or symptoms of diabetes, except for weight loss (*p* = 0.032). The correlation was poor between symptoms of hyperglycaemia and abnormal glucose testing probably because the majority of these participants were prediabetic rather than diabetic, having mild hyperglycemia. This reveals the need for glycemic screening in asymptomatic high-risk individuals.

A potential limitation of studies in pediatric subjects and adolescents could be difficulties in achieving a good response rate with the risk of sampling bias. Letters of invitation were sent to consent parents on using their children’s data. Those who did not respond were sent further second and third reminders. If still not responding the wish of parents was respected and other details of the student were not captured (blood pressure, glycemic testing etc.). We were not able to identify anything distinctive for those children and adolescents concerning age, gender and BMI and we don’t have further data in able to analyze the characteristics of non-respondents in comparison with respondents. In our study the response rate was 72 % at the first invitation and 79 % in the second phase (Fig. 1). All though we cannot be sure about the student’s reason for not participating in the study and there might be a possibility of selection bias, we assume the main reason to be unwillingness to be exposed to finger pricking.

## Conclusion

This study shows a worrying high proportion of prediabetes and diabetes among Emirati overweight and obese children and adolescents based on fasting blood glucose and/or OGTT. These results are not consistent with the results based on the HbA1c method. Diagnostic screening practices including fasting blood glucose and/or OGTT indicated a lower prevalence of prediabetes compared to HbA1c screening. The correlation between capillary HbA1c values showed poor agreement with capillary fasting blood glucose values. Overall adiposity (BMI Z-score), family history of T2D first-degree relatives and high levels of triglycerides was statistically significant correlated with abnormal glycemic testing. A follow-up study is needed to clarify which of the methods would be deemed most reliable in predicting progression to diabetes among prediabetes children and adolescents.

## Abbreviations

ADA: American Diabetes Association
BP: blood pressure
BMI: body mass index
CDC: Center for Disease Control and Prevention
DBP: diastolic blood pressure
FBG: fasting blood glucose
HbA1c: glycated hemoglobin
HDL: high-density lipoprotein
LDL: low-density lipoprotein
OGTT: oral glucose tolerance tests
RCDR: Rashid Centre for Diabetes and Research
SBP: systolic blood pressure
T2D: type 2 diabetes
UAE: United Arab Emirates
WC: waist circumference
